# Characteristics of microcrystalline cellulose derived from oil palm trunk slabs and its potential for use as tablet diluent

**DOI:** 10.1016/j.heliyon.2025.e42902

**Published:** 2025-02-21

**Authors:** Chutima Jantarat, Sirikanya Kaewpradit, Jiraporn Chingunpitak, Suthon Srivaro

**Affiliations:** a Drug and Cosmetics Excellence Center, Walailak University, Thasala, Nakhon Si Thammarat, 80160, Thailand; b School of Pharmacy, Walailak University, Thasala, Nakhon Si Thammarat, 80160, Thailand; c Center of Excellence in Wood and Biomaterials, School of Engineering and Technology, Walailak University, Nakhon Si Thammarat, 80160, Thailand

**Keywords:** Microcrystalline cellulose, Pharmaceutical excipient, Oil palm trunk slabs, Tablet diluent

## Abstract

This work aimed to investigate the potential of cellulose extracted from oil palm trunk slabs (OPTS), residues from oil palm lumber processing for pharmaceutical tablets. Due to varying wood cell characteristics within the oil palm trunk which might affect the quality of the cellulose extracted from OPTS, the suitability of cellulose extracted from different positions within OPTS with respect to trunk height for tablet diluent was therefore explored in this work. OPTS from the trunk's bottom, middle, and top parts were collected. The vascular bundle and parenchyma tissue of each OPTS section were then mechanically separated and converted into powders. Cellulose was extracted from the powder and microcrystalline cellulose (MCC) was derived using acid hydrolysis. The powder and tablet compression properties of the MCC were then evaluated and compared with that of the commercial ones. MCC yields from both types of wood cells decreased from the bottom to the top of the trunk. MCC obtained from parenchyma tissue was more suitable for pharmaceutical tablets due to its finer texture and smaller particle size, and only parenchyma-derived MCC was used for further study; except MCC from the top part was ignored due to its relatively low MCC yield. Most of the parenchyma-derived MCC's properties obtained from the bottom part were greater than that of the middle part but slightly lower than that of the commercial MCC, Comprecel® M102. The study of tablet compression properties of the MCC revealed that the tablet prepared from the MCC of the bottom part met the general requirement for tablet manufacturing, indicating that it could be potentially used as tablet diluents.

## Highlights

• The utilization of oil palm trunk slabs is being explored for pharmaceutical tablets.
• Microcrystalline cellulose (MCC) derived from oil palm trunk slabs is characterized.
• Properties of MCC from different parts and cell types of the trunk are different.
• MCC from parenchyma tissue has superior properties than that of vascular bundles.
• MCC from parenchyma tissue of bottom part has potential for use as tablet diluent.

## Graphical abstract

**Figure:**
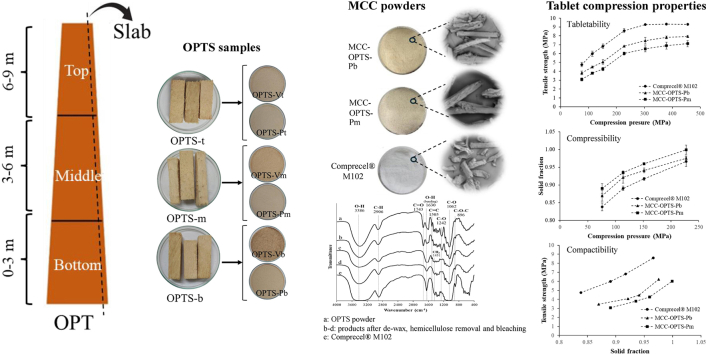

## 1 Introduction

Oil palm (*Elaeis guineensis*) is considered an important economic crop in the tropics, especially in Asia. Thailand has the 3^rd^ largest oil palm plantation area in the world after Indonesia and Malaysia [1]. Although the oil palm industry drives economic growth, it also produces significant amounts of agricultural waste. The oil palm trunk (OPT) represents a substantial quantity of solid waste produced when oil palm trees are felled [2]. Recently, the use of oil palm trunk lumber for furniture and building materials has been confirmed [[3], [4], [5]]. However, OPT slabs (OPTS), the outermost portion of OPT, generated during lumber production are still left as waste material and typically disposed of by burning. As cellulose is the main component of the oil palm trunk [6], the utilization of cellulose from OPTS for pharmaceutical applications is of interest in this work.

Plant cellulose has been widely used as a diluent in tablet formulation due to its compatibility with human tissue and good mechanical resistance to compressive forces during forming the tablet [7,8]. The diluent is generally considered as the most important part of tablet formulation due to its high proportion in the overall content, and it determines the dissolution and disintegration properties of the tablet [9,10]. Preparation of plant cellulose for use as diluent consists of two main steps. Firstly, cellulose is isolated from hemicellulose and lignin using the solvent method. The amorphous region of the cellulose is then removed using either chemical or enzymatic processes [11,12], resulting in microcrystalline cellulose (MCC) for use as diluent. The characteristic of MCC is dependent on various factors such as the type of plant cellulose, isolation process of cellulose, pretreatment, etc. [[13], [14], [15]].

Few studies have reported the isolation and characterization of cellulose from oil palm trunks to prepare cellulose nanocrystals (CNC) or microcrystalline cellulose (MCC) [[16], [17], [18], [19]]. From the previous studies [16], it was reported that the pretreatment of raw OPT with hot water before the cellulose isolation process reduced the production yield of cellulose by approximately 5 % compared to the toluene/ethanol pretreatment while the cellulose's properties remained the same. In addition, it was also reported that modifying cellulose extracted from oil palm trunk with acid hydrolysis resulted in increased crystallinity of cellulose up to 57–75 % [17,19]. Moreover, the characteristics of CNC derived from parenchyma tissue and vascular bundles have also been reported. It was found that CNC obtained from parenchyma tissue had smoother and cleaner surface and higher thermal stability, but CNC obtained from vascular bundles had higher crystallinity index [17]. However, the effect of the position of wood within a trunk on the isolation process and characteristics of cellulose has not been reported. With the varying amounts and characteristics of fibers and parenchyma cells throughout its cross section and the trunk's height, the wood position could become a potential parameter affecting the isolation process and characteristics of cellulose from OPTS studied herein.

This study aimed to investigate the feasibility of MCC derived from OPTS cellulose, a sustainable source of cellulose raw material, for use as a diluent in pharmaceutical tablets. OPTS from three different positions along the trunk's height namely the bottom, middle, and top parts were collected for the experiment. The vascular bundles and parenchyma tissue were mechanically separated and grounded into powder. The cellulose was then extracted using a simple chemical method before being derived into MCC using acid hydrolysis. The properties of the obtained MCC were then measured, and compared with the commercial MCC, Comprecel® M102.

## 2 Materials and methods

### 2.1 Materials

Oil palm trunk slabs (OPTS) used as a cellulose source were obtained from the 25 years old oil palm tree with the height of approximately 9 m from the plantation area in Thasala district, Nakhon Si Thammarat province, Thailand. Different OPTS sections: bottom (OPTS-b), middle (OPTS-m), and top (OPTS-t) were collected from different OPT sections according to the height from the ground: 0–3 m, 3–6 m, and 6–9 m, respectively. Comprecel® M102, a commercial microcrystalline cellulose (MCC), used as a reference material for comparison in this work, was purchased from Mingtai Chemical Co. Ltd. (Taiwan). All chemicals and solvents used were of analytical grade, consisting of potassium hydroxide (KOH), toluene, ethanol (95 %), and sulfuric acid (H_2_SO_4_) purchased from RCI Labscan Limited (Bangkok, Thailand), and hydrogen peroxide (30 %) purchased from Sigma-Aldrich Chemie (Steinheim, Germany).

### 2.2 Preparation of MCC from OPTS

**Fig. 1:**
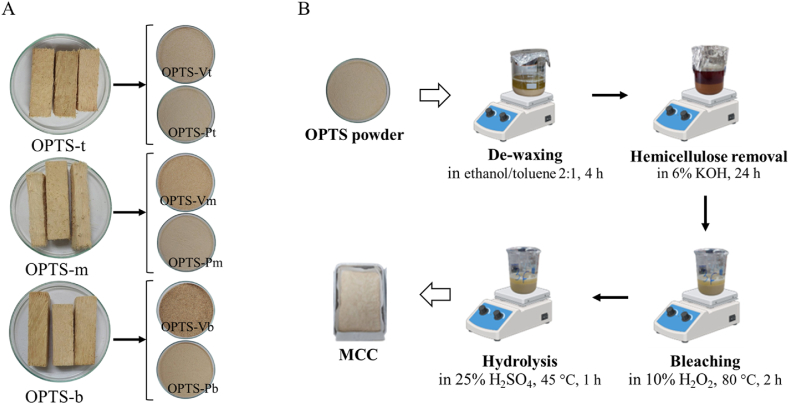
A: Appearance of OPTS pieces and powder of each part to be used as a source for MCC preparation; B: Procedure for preparing MCC from OPTS powder.

Three different OPTS sections: OPTS-b, OPTS-m, and OPTS-t, were cut into small pieces approximately 2 cm × 2 cm × 5 cm, then washed of dust and dirt with distilled water and dried at 60 °C until a constant weight was obtained. OPTS was grounded into a coarse powder using a cutting mill (Retsch SM 100, Haan, Germany) with a sieve size of 8 mm. Vascular bundles and parenchyma tissue of each OPTS section were separated mechanically. Each sample was then ground into a fine powder using a hammer mill (Polymix® PX-MFC 90 D, Kinematica, Malters, Switzerland) with a sieve size of 2 mm. Three parts of vascular bundle powder: OPTS-Vb, OTPS-Vm, and OPTS-Vt, and three parts of parenchyma tissue powder: OPTS-Pb, OPTS-Pm, and OPTS-Pt were obtained from OPTS-b, OPTS-m, and OPTS-t, respectively. The appearance of OPTS pieces and OPTS powder, both vascular bundle and parenchyma tissue powders, is shown in Fig. 1A. All sample powders were subjected to cellulose extraction using an identical procedure adopted from the method of Lamaming et al. [18], which consisted of three main steps: de-waxing, hemicellulose removal and bleaching (Fig. 1B), as follows. OPTS powder (80 g) was soaked in 400 mL of ethanol-toluene mixture (ratio 2:1) and stirred at 500 rpm for 4 h to remove raw wax from OPTS powder. After drying the OPTS powder, hemicellulose removal was performed by immersing the OPTS powder in 600 mL of 6 % (w/v) KOH solution and stirring at 500 rpm for 24 h. The obtained cellulose was washed with distilled water in a solid/liquid ratio of 1:4 by centrifugation 3 times. Finally, the obtained cellulose was bleached to remove lignin and other residual contaminants by immersing in 10 % (w/v) of H_2_O_2_ in a solid/liquid ratio of 1:4 at 80 °C under continuous stirring at 500 rpm for 2 h. The obtained cellulose was washed with distilled water in a solid/liquid ratio of 1:4 by centrifugation 5 times.

To transform cellulose into MCC, sulfuric acid, which has been confirmed to be effectively used for hydrolysis of cellulose to MCC [15,18,20], with a concentration of 25 % (based on the preliminary result) was used to hydrolyze cellulose from all parts of the oil palm trunk (bottom, middle, and top). The reaction was carried out in a solid/liquid ratio of 1:4 at 45 °C under continuous stirring at 500 rpm for 1 h and stopped by soaking with cool water. The obtained MCCs which included MCC-OPTS-Vb, MCC-OPTS-Vm, and MCC-OPTS-Vt from vascular bundle part, and MCC-OPTS-Pb, MCC-OPTS-Pm, and MCC-OPTS-Pt from parenchyma tissue part of OPTS-b, OPTS-m, and OPTS-t, respectively were washed with distilled water in a solid/liquid ratio of 1:4 by centrifugation until the pH of the washed water was close to 7. The obtained MCC was dried in the hot air oven at 55 °C for 24 h and then sieved through a sieve with an appropriate mesh size of No. 40. The yield of the product obtained at each step of the MCC preparation process was calculated using Equation (1).

(1) $$\text{Yield}\;\text{of}\;\text{product}\;\left(\%\right)=\frac{\text{Weight}\;\text{of}\;\text{product}\;\text{obtained}\;\left(g\right)}{\text{Weight}\;\text{of}\;\text{OPTS}\;\text{powder}\;\left(g\right)}\times 100$$

### 2.3 Particle and powder characterization

#### 2.3.1 Fourier transform infrared (FTIR) spectroscopy

FTIR was used to determine the functional structure of the products obtained from each step of the MCC preparation process. The sample powder was mixed with KBr in an amount of approximately 100 mg to achieve a concentration of 1 % sample powder in KBr. Then, the solid mixture was pressed at 53.4 kN using a hydraulic press machine to obtain a sample disc before being analyzed with FTIR (Tensor 27; Bruker, Ettlingen, Germany). The wavenumber region was in the range of 4000–400 cm^−1^, and the resolution was 4 cm^−1^. Comprecel® M102 was used as the reference MCC for comparison.

#### 2.3.2 Scanning electron microscopy (SEM)

SEM was used to determine the morphology of MCC obtained from different OPTS sections. The sample particles were coated with a thin layer of gold by a sputter coater (108Auto; Cressington, Watford, UK). The coating current was set to 20 mA with a sputtering time of 180 s. Then, the obtained samples were examined by SEM (Zeiss Merlin FEG-SEM; Carl Zeiss, Oberkochen, Germany). Comprecel® M102 was used as the reference MCC for comparison.

#### 2.3.3 X-ray powder diffraction (XRD)

XRD was used to determine the crystallinity of cellulose and MCC obtained from different OPTS sections. Two grams of the powder sample were weighed and analyzed by an X-ray diffractometer (Empyrean; Panalytical, Netherlands). A voltage of 40 kV at 30 mA was used. The samples were scanned within the 2θ range of 5–90° with a step size of 0.026°. From the diffraction profile, the percent crystallinity was determined between the ratio of area under the peaks of crystalline diffracted to the total area under the curve as shown in Equation (2).

(2) $$\text{Crystallinity}\;\left(\%\right)=\frac{S_{C}}{S_{C}+S_{A}}\times 100$$

Where *S*_*C*_ is the area under the crystalline peaks and *S*_*A*_ is the area under the amorphous peaks.

#### 2.3.4 Flowability

The flowability of the prepared MCC powder was evaluated according to general chapter <1174> powder flow of the United States Pharmacopeia (USP) 2023 [21] compared with Comprecel® M102. Each sample was analyzed concerning three parameters: angle of repose, compressibility index, and Hausner ratio. To determine the angle of repose, the powder was filled onto the upper portion of the funnel while the height of the terminal funnel from the base remained constant at 10 cm. The powder was allowed to flow freely to the base, and then the height and diameter of the cone shape of the powder falling on the base were measured. The angle of repose (σ) was calculated using Equation (3).

(3) $$\tan\;\sigma =\frac{height}{0.5\;\text{base}}$$

The compressibility index and Hausner ratio were calculated from the bulk density (ρ_bulk_) and tapped density (ρ_tapped_) of the powder*.* The sample was weighed (20 g) and poured into a 100 mL cylinder. The bulk density (ρ_bulk_) and tapped density (ρ_tapped_) were then measured from the untapped volume and tapped volume, respectively, according to general chapter <616> bulk density and tapped density of powders of the USP 2023 [22]. The compressibility index and Hausner ratio were calculated using the following Equations:

(4) $$\text{Compressibility}\;\text{index}=\left(\frac{\rho_{tapped}-\rho_{bulk}}{\rho_{tapped}}\right)\times 100$$

(5) $$\text{Hausner}\;\text{ratio}=\frac{\rho_{tapped}}{\rho_{bulk}}$$

#### 2.3.5 True density

The true density of the prepared MCC powder and Comprecel® M102 was determined using a helium pycnometer (AccuPyc II 1340, Micromeritics, GA, USA) on 10 mg of sample.

#### 2.3.6 Degree of polymerization

The degree of polymerization of the prepared MCC powder and Comprecel® M102 was determined according to the method described in the USP Microcrystalline cellulose monograph [23]. The samples were dissolved in 0.5 M bis(ethylenediamine)-copper solution to achieve a concentration of 1.3 g/50 mL. Then, the viscosity of samples was determined through a 150 mL Cannon-Fenske viscometer recording the flow times in terms of t_1_ as described in Equation (6). The viscosity of bis(ethylenediamine)-copper solution as the blank solution was measured through a 100 mL Cannon-Fenske viscometer and recorded the flow times in terms of t_2_ as described in Equation (7). The relative viscosity (η_rel_) of each sample to blank was calculated as described in Equation (8). The intrinsic viscosity ([η]_c_) was obtained from the USP intrinsic viscosity table. Then, the degree of polymerization (DP) was calculated using Equation (9).

(6) $${\text{KV}}_{1}=t_{1}\times k_{1}$$

(7) $${KV}_{2}=t_{2}\times k_{2}$$

(8) $$\eta_{rel}=\frac{{KV}_{2}}{{KV}_{2}}$$

(9) $$\text{DP}=\frac{95\times {\left[\eta \right]}_{c}}{WS\times \frac{100-\%LOD}{100}}$$

Where KV_1_ is kinematic viscosity of sample, k_1_ is viscometer constant of 150 mL Cannon-Fenske viscometers, t_1_ is flow times of sample, KV_2_ is kinematic viscosity of blank, k_2_ is viscometer constant of 100 mL Cannon-Fenske viscometers, t_2_ is flow times of blank, η_rel_ is relative viscosity, [η]_c_ is intrinsic viscosity used from USP intrinsic viscosity table, DP is degree of polymerization, WS is weight of cellulose (g), and %LOD is the percentage loss on drying.

#### 2.3.7 Inorganic impurities

The inorganic impurities of the prepared MCC powder and Comprecel® M102 were determined according to the residue on ignition method [24]. Five grams of sample were placed in a tarred porcelain crucible, moistened with 1–2 mL H_2_SO_4_, and heated until white fumes no longer evolved. The crucible was then ignited at 600 ± 50 °C for 30 min, cooled in a desiccator, and weighed to determine the remaining amount of substance.

#### 2.3.8 pH

The prepared MCC powder and Comprecel® M102 (5 g each) were shaken with 40 mL of distilled water for 20 min and then centrifuged. The supernatant was used for pH measurement.

#### 2.3.9 Loss on drying (LOD)

The moisture contained in the prepared MCC powder and Comprecel® M102 was carried out by a loss on drying (LOD) test. A 3 g sample was used for determination using a moisture analyzer (HR83, Mettler-Toledo, Greifenesee, Switzerland). LOD was calculated according to the formula below:

(10) $$\text{LOD}\;\left(\%\right)=\frac{W_{0}-W_{1}}{W_{0}}\times 100$$

where W_0_ and W_1_ are the weight of the initial sample and after drying to constant weight, respectively.

#### 2.3.10 Water soluble substances

The prepared MCC powder and Comprecel® M102 (5 g each) were shaken with 80 mL distilled water for 10 min, then filtered through filter paper. The filtrate was dried at 105 °C for 1 h, allowed to cool in a desiccator, and weighed to determine the remaining amount of substance.

#### 2.3.11 Ether soluble substances

The prepared MCC powder and Comprecel® M102 (10 g each) were placed in a glass syringe (25 mL) and 50 mL of ether was passed through the syringe. The obtained eluate was dried in a fume hood followed by drying at 105 °C for 30 min, cooled in a desiccator, and weighed to determine the remaining amount of substance.

### 2.4 Tablet compression studies

#### 2.4.1 Tabletability analysis

The relationship between the compression pressure and the tensile strength of the MCC derived from different sections of OPTS was studied in comparison with Comprecel® M102. The MCC powder (300 mg each) was pressed to a tablet using a hydraulic press (Press-200, Panchum Scientific Corp, Kaohsiung, Taiwan) with a 13 mm flat punch at different compression pressures ranging between 75 and 455 MPa. The tablet breaking force, thickness, and diameter were measured using the tablet hardness, thickness, and diameter tester (PTB511, Pharma Test Apparatebau AG, Hainburg, Germany). The tensile strength was calculated from the breaking force and tablet dimension as following Equation:

(11) $$T\text{ensile}\;\text{strength}\;\left(\text{MPa}\right)=\frac{2F}{\pi DT}$$

Where *F* is the breaking force (N), *D* is the diameter of the tablet (mm), and *T* is the tablet thickness (mm). The tabletability profile of each MCC was plotted between the compression pressure and the tensile strength.

#### 2.4.2 Compressibility analysis

The relationship between compression pressure and solid fraction called compressibility of MCC obtained from different OPTS sections was studied in comparison with Comprecel® M102. The solid fraction is the ratio of the tablet density and the true density of uncompressed powder (Equation (12)). The tablet density was calculated from its weight and volume. The compressibility profile of each MCC was plotted between the compression pressure and the solid fraction.

(12) $$S\text{olid}\;\text{fraction}=\frac{\text{Tablet}\;\text{density}}{\rho_{true}\;\text{of}\;\text{uncompressed}\;\text{powder}}$$

#### 2.4.3 Compactibility analysis

The relationship between the solid fraction and the tensile strength called compactibility of MCC obtained from different OPTS sections was also studied in comparison with Comprecel® M102. The compactibility profile of each MCC was plotted between the solid fraction and the tensile strength.

#### 2.4.4 Elastic recovery determination

The elastic recovery of tablets prepared from MCC obtained from different OPTS sections was determined in comparison with tablets prepared from Comprecel® M102 by calculating the initial tablet thickness and the tablet thickness at 24 h after compression as described in Equation (13).

(13) $$\text{Elastic}\;\text{recovery}\;\left(\%\right)=\frac{H_{1}-H_{0}}{H_{0}}\times 100$$

Where *H*_*1*_ is the thickness of the tablet at 24 h after compression, and *H*_*0*_ is the thickness of the same tablet at the initial time. Then, the elastic recovery profile of each MCC was plotted between the elastic recovery percentage and compression pressure.

## 3 Result and discussion

### 3.1 Preparation and yield of MCC derived from OPTS

MCC was successfully prepared from OPTS after using three steps for cellulose extraction: de-waxing with ethanol/toluene, hemicellulose removal by KOH, and bleaching with H_2_O_2_, followed by acid hydrolysis using H_2_SO_4_. The preparation method was initially attempted from the method reported by Lamaming et al.’s research group [18] for the extraction of cellulose from oil palm trunks (OPT), but some steps were modified to make them more efficient for this study. Since the objective of this study was to compare the properties of MCC obtained from different OPTS sections, it was necessary to use the same preparation method performed on all OPTS sections and make it more practical. Therefore, in the bleaching step, H_2_O_2_ was used instead of sodium chlorite (NaClO_2_), and the step was changed to perform after hemicellulose removal. NaClO_2_ is widely used in the process of extracting cellulose from plants to remove lignin [25]. Generally, approximately 1 % NaClO_2_ was reported to be used in the extraction of oil palm cellulose [15,[26], [27], [28]]. However, in the preliminary study of this study, NaClO_2_ at 1 % was found to have no significant effect on lignin removal. This might be due to the use of too low a concentration of NaClO_2_. Effective removal of lignin using NaClO_2_ required NaClO_2_ concentrations as high as 8 % [29]. However, due to environmental and safety concerns, chlorine-free cellulose extraction from plants should be considered instead. H_2_O_2_ was used in this study instead of NaClO_2_. Although H_2_O_2_ has a lower redox potential than NaClO_2_, the redox potential of H_2_O_2_ can increase with temperature [30]. In the bleaching process with H_2_O_2_ in this study, the temperature was therefore set at 80 °C. This temperature was considered sufficient to increase the redox potential of H_2_O_2_ for bleaching because it can facilitate the production of hydroxyl radicals (OH•), which can destroy the structure of lignin [30].

In this study, the bleaching process was performed after hemicellulose removal by alkaline treatment to make it more efficient and practical. Alkaline condition is another factor that can enhance the redox potential level of H_2_O_2_ from increased hydroxyl radicals (OH•) [31]. In addition, the removal of hemicellulose by alkaline treatment can also remove lignin [32]. Even though the compound obtained by removing hemicellulose was washed with water 3 times before bleaching, the pH of the washing medium remained alkaline (pH approximately 10). It should therefore be beneficial to bleach with H_2_O_2_ after hemicellulose removal because alkaline adjustment is not required before processing. Since most of the lignin was removed from the hemicellulose removal process (as obtained from FTIR monitoring), H_2_O_2_ could work effectively to remove residual lignin in the bleaching process. In addition, according to its mechanism of action, H_2_O_2_ can remove other residual contaminants. Therefore, the H_2_O_2_ bleaching sequence as the final step in cellulose preparation should ensure the high purity of the obtained cellulose.

The reaction time for bleaching must also be considered because hydroxyl radical (OH•) liberated from H_2_O_2_ has low reaction selectivity for lignin and therefore can react with cellulose as well, which would reduce the cellulose yield [30]. It was found that a reaction time of 2 h was appropriate because it resulted in cellulose with an acceptable color (off-white) and yield (average yield of approximately 44 % from all OPTS parts, which decreased by approximately 3 % from the hemicellulose removal process).

OPT has a variety of properties along both radial and length directions [33]. That was the reason for this study to compare the properties of cellulose obtained from different sections of OPTS. OPT consists of two main parts: parenchyma and vascular bundle. These two parts have different physical characteristics: the parenchyma is soft and spongy, while the vascular bundle is dense and fibrous, which can be segregated by pounding [17]. In a previous study, the vascular bundle and parenchyma were separated before cellulose was extracted from each part [17]. When OPTS was ground into a coarse powder, vascular bundles, and parenchyma could be easily separated by a sieve with an appropriate mesh size of No. 40.

**Fig. 2:**
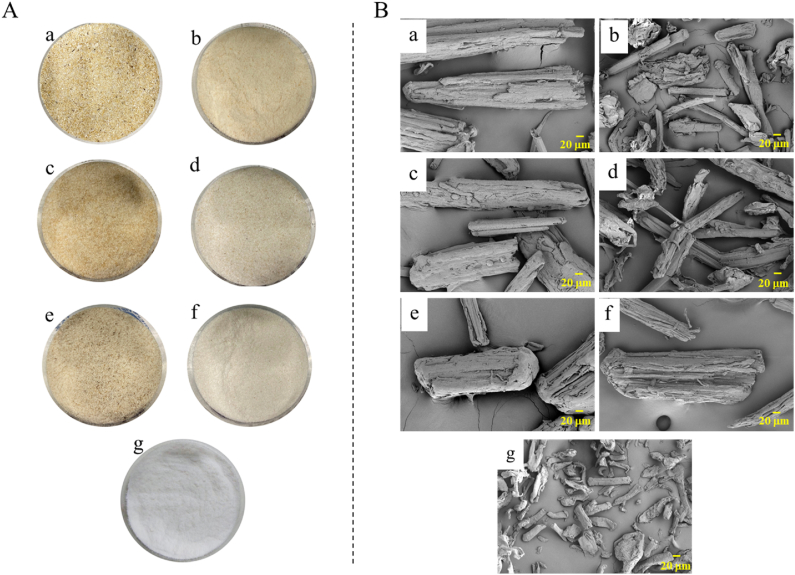
A: Picture and B: SEM images of MCC obtained from different sections and parts of OPTS (a, c, and e − MCC from OPTS-Vb, OPTS-Vm, and OPTS-Vt, respectively; b, d, and f – MCC from OPTS-Pb, OPTS-Pm, and OPTS-Pt, respectively; g – Comprecel® M102).

Table 1 shows the product yields obtained at each step of MCC preparation from different OPTS parts. The de-waxing process affected OPTS-Vb and OPTS-Vm more than other parts. This might be because most of the raw wax is assembled around the vascular bundle in the lower part of the OPT due to age. The yield changed significantly after alkaline treatment in the hemicellulose removal process. This is because high concentration of alkaline solution (6 % KOH) can remove hemicellulose by cleaving the ester linkages between hemicelluloses and cellulose, then removing the hemicelluloses’ part from the cellulose [34]. Alkaline treatment also affects the removal of lignin, thus disrupting the connection between the three components: hemicellulose, lignin, and cellulose [35]. Hemicellulose and lignin were dissolved and removed by washing the resulting insoluble cellulose with water. The yield of the product obtained from the three vascular buddle powder samples after hemicellulose removal was not much different, they were about 50 %. However, there were high differences among the parenchyma powder samples. The highest yield obtained from OPTS-Pb was 52.75 % and decreased accordingly with the height of OPT from OPTS-Pm (30.85 %) and OPTS-Pt (9.40 %), respectively with the average yield was 31 %. These results indicate that the cellulose content in the vascular bundle was higher than in the parenchyma, which is consistent with a previous report [36], and that the cellulose content decreases with the OPT height from the ground due to age, which is associated with a decrease in density with the OPT height from bottom to top [33]. In addition to cellulose, hemicellulose, and lignin, OPT contains other components, especially starch and sugars. It was reported that OPT contained starch as much as 55.5 % [37]. In the parenchyma, especially in the upper part of OPTS, there tends to be a much higher content of starch and sugars than cellulose, which can be dissolved and removed by alkaline treatment, therefore the yield of obtained cellulose is relatively low. In the bleaching process, the product yields obtained from all OPTS parts were not much changed (1–3 %) from the previous process. This would be because in this process only residual lignin and other residual contaminants would be removed to obtain clean cellulose. As a result, the color of the product powder visually changed from brown to off-white (as shown in Fig. 2A). The total cellulose obtained from OPTS-b and OPTS-m was approximately 44 %, which is slightly higher than the cellulose content in OPT previously reported (approximately 41 %) [18]. This would be related to the fact that the OPTS has a higher density than the internal parts of OPT due to its higher cellulose content [33].

**Table 1:** Product yields obtained in each process from different OPTS parts.

Process	Product yield (%)					
	OPTS-Vb	OPTS-Vm	OPTS-Vt	OPTS-Pb	OPTS-Pm	OPTS-Pt
De-waxing	85.84 ± 2.54	88.70 ± 2.26	94.48 ± 1.98	98.75 ± 0.47	97.80 ± 0.78	95.35 ± 0.91
Hemicellulose removal	53.48 ± 1.36	48.17 ± 1.24	46.10 ± 1.32	52.75 ± 1.08	30.85 ± 1.01	9.40 ± 0.82
Bleaching	50.69 ± 1.19	46.22 ± 1.05	44.14 ± 0.94	50.81 ± 0.72	28.27 ± 1.10	8.62 ± 0.22
Acid hydrolysis	48.04 ± 0.67	43.29 ± 1.11	40.49 ± 0.55	33.80 ± 0.91	18.5 ± 1.06	8.35 ± 0.50

In the final process, acid hydrolysis to transform cellulose into MCC, the product yields in the fine powder samples decreased significantly, while the change in the vascular bundle powder samples was relatively low. The cellulose chains from the parenchyma should have both crystalline and amorphous regions, giving a soft texture, while the cellulose chains from the vascular bundle likely had an almost entirely crystalline form, giving a rough texture. Acid hydrolysis therefore affected the yield of cellulose products obtained from the parenchyma powder samples, especially OPTS-Pb and OPTS-Pm, because amorphous regions in the cellulose chains were removed. The average MCC yield obtained for vascular bundle samples was approximately 44 %, while for the parenchyma samples, the average MCC yield was approximately 20 %; This is not much different from the previously reported MCC obtained from OPT of 23–28 % [16].

### 3.2 Appearance and morphology of MCC derived from OPTS

The appearance of MCC prepared from different OPTS parts is shown in Fig. 2A. The texture of MCC obtained from vascular bundle samples was relatively rough and contained relatively large particles, while the MCC obtained from parenchyma samples was relatively fine and had relatively smaller particles. This is consistent with the fact that the physical characteristics of parenchyma are soft and spongy, while vascular bundles have a dense, fibrous texture and are less hygroscopic [17]. The texture of MCC obtained from parenchyma samples, especially from OPTS-Pb was similar to the texture of the commercial MCC, Comprecel® M102. The color of vascular bundle MCC was a darker brown than the color of parenchyma MCC, which was off-white, while Comprecel® M102 was white. For the parenchyma MCC obtained, the brown color gradually faded as the OPTS was used as a source from bottom to top. The color of MCC products was related to the color of the initial OPTS powder of each part. The bleaching process could reduce the color of cellulose from brown to off-white, which was achieved from only a one-time bleaching process with all parts being the same. The cellulose obtained from the lower part was darker, probably due to the natural aging of the cell walls.

The morphology of MCC obtained from different OPTS parts is shown in Fig. 2B. The size and shape of the MCC obtained from the vascular bundle samples were relatively the same, with a larger rod shape than the MCC obtained from the parenchyma samples. As for the parenchyma MCC, they also had the shape of a rod, but their size was different, being small and increasing sequentially from bottom to top OPTS. The estimated lengthwise dimensions from SEM images were (mean ± S.D., n = 10 each) 161.26 ± 29.15, 273.77 ± 53.83, 311.17 ± 88.57 μm for the parenchyma MCC from bottom, middle, and top OPTS, respectively, and 562.10 ± 44.45, 547.14 ± 80.27, 463.52 ± 51.49 μm for the vascular bundle MCC from bottom, middle, and top OPTS, respectively, while 92.40 ± 26.75 μm for Comprecel® M102. MCC obtained from OPTS-Pb, which had the smallest size, had the most similar morphology (size and shape) to Comprecel® M102.

### 3.3 FTIR

**Fig. 3:**
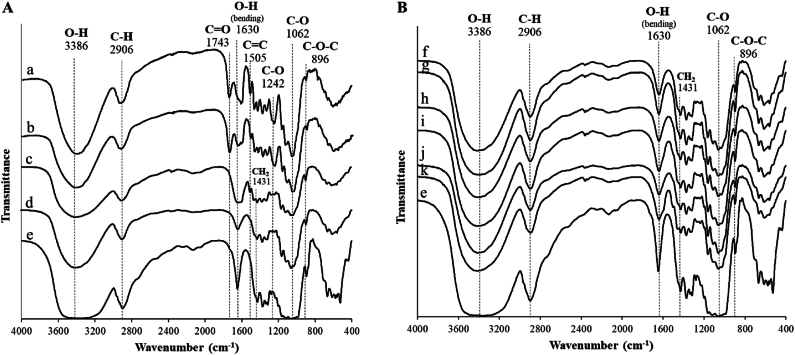
FTIR spectra of A: products obtained during the MCC preparation process by OPTS-Pb shown as representative, a, b, c, and d are OPTS powder, products after de-wax, hemicellulose removal, and bleaching process, respectively compared to (e) Comprecel® M102; and B: MCC obtained from different OPTS parts, f, g, and h are OPTS-Vb, -Vm, and -Vt, respectively and i, j, and k are OPTS-Pb, -Pm, and -Pt, respectively compared to (e) Comprecel® M102.

By following the chemical changes of OPTS cellulose and MCC obtained from OPTS during the MCC preparation process by FTIR, Fig. 3 shows FTIR spectra of products obtained during the MCC preparation process (Fig. 3A) and MCC obtained from different OPTS parts (Fig. 3B), respectively. As shown in Fig. 3A, the spectrum of each process exhibited the main key component of cellulose consisting of the hydroxy group (O-H) as the broad shape at around 3386 cm^−1^, alkane group (C-H) which is the main structure of cellulose at around 2906 cm^−1^, the hydrogen-bonded hydroxyl group (O-H bending) of cellulose and absorbed water at around 1630 cm^−1^, the ether group (C-O) at around 1062 cm^−1^, and the ether group (C-O-C) at around 896 cm^−1^ which indicated the band of β-glycosidic linkages [38]. The obtained cellulose spectrum (Fig. 3A–d) was similar to that of Comprecel® M102 (Fig. 3A–e). These results indicate that the preparation process used to prepare cellulose in this study did not destroy the cellulose structure during cellulose preparation. OPTS showed a band at around 1743 cm^−1^ which was attributed to xylan, which is a hemicellulose [39]. OPTS also had a band around 1505 cm^−1^, which was attributed to C-O stretching in lignin [39], and a band around 1242 cm^−1^, which was attributed to C-O stretching in lignin and xylan [38]. The peaks attributed to the functional groups of xylan and lignin decreased or disappeared after alkaline treatment in the hemicellulose removal process. The alkaline solution can extract xylan from OPT composition [37]. Lignin can be also removed by alkaline treatment [37]. The bleaching process can also remove both hemicellulose and lignin [32]. Therefore, after the bleaching process, the resulting cellulose showed a sharper peak at around 1630 cm^−1^ and the peak disappeared completely at around 1505 cm^−1^ due to the removal of residual lignin and other residual contaminants.

When the obtained cellulose was further transformed into MCC by acid hydrolysis, the MCC obtained from all OPTS parts showed the same spectral pattern as cellulose and Comprecel® M102 (Fig. 3B), indicating that this process was successfully to prepare MCC from any OPTS part. The peak at around 1430 cm^−1^, which corresponds to the crystallinity of the compound [40], was found to increase slightly after acid hydrolysis. This is likely due to the removal of amorphous regions from the cellulose chains by acid hydrolysis.

### 3.4 XRD

**Fig. 4:**
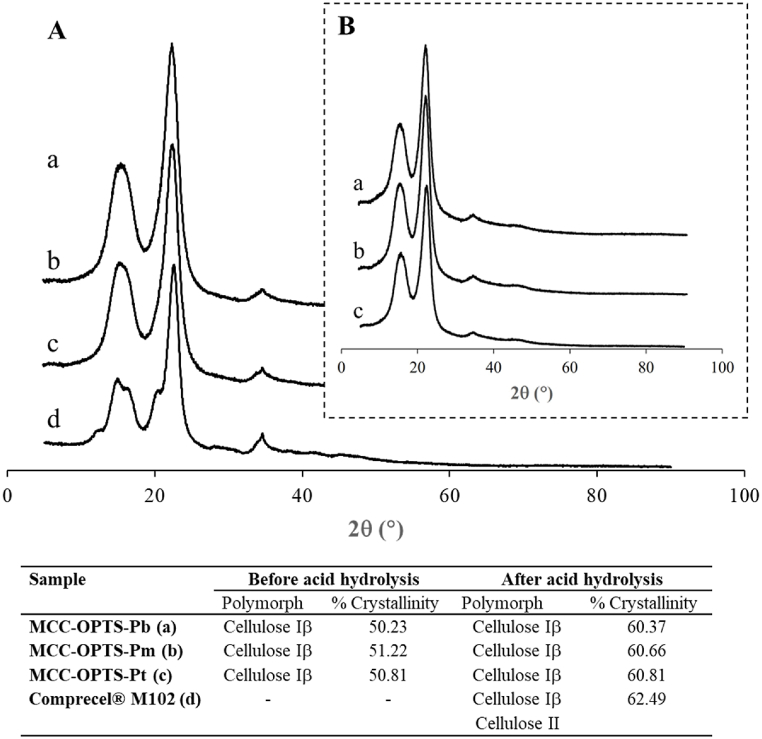
XRD patterns of A: MCC obtained from (a) OPTS-Pb, (b) OPTS-Pm, and (c) OPTS-Pt after hydrolysis compared to (d) Comprecel® M102, and compared to B: cellulose before acid hydrolysis.

Using XRD to evaluate the crystalline behavior of cellulose and MCC obtained from OPTS following the MCC preparation process, the results are shown in Fig. 4. The obtained cellulose (before acid hydrolysis) and the MCC obtained from parenchyma parts of OPTS of all three sections (after acid hydrolysis) showed main peaks at around 2θ = 15.5°, 22.5°, and 34.6°, indicating cellulose polymorph of cellulose Iβ, while Comprecel® M102 showed an additional peak at around 12.3°, 14.8°, 16.2°, and 20.4°, indicating a structure composed of both cellulose Iβ and II [41]. The structure of cellulose I can be changed to cellulose II by alkaline treatment [42], however, the concentration of alkaline treatment in this study (6 % KOH) might not be high, resulting in the prepared MCC containing only cellulose Iβ. The XRD patterns of cellulose before and after acid hydrolysis were similar, which means that the hydrolysis condition in this study did not affect the polymorphism of OPTS-derived cellulose converted to MCC. The crystallinity of OPTS-derived cellulose was approximately 50 %, which should be higher than the crystallinity of raw OPTS powder, where the crystallinity of OPT powder was reported to be approximately 37 % [38]. After acid hydrolysis of cellulose into MCC, the resulting crystallinity of MCC increased to about 60 %. This was because the acid hydrolysis condition used in this study could remove some amorphous regions from the cellulose chains. It should be noted that the crystallinity of the obtained MCC was slightly lower (approximately 2 %) compared to the commercial MCC Comprecel® M102, which was used as a reference material. One important factor affecting the percentage of crystallinity is the acid concentration. Acid hydrolysis of cellulose materials results in the decomposition of amorphous regions, which affects the percentage of crystallinity of the obtained cellulose [43]. The reported acid concentration used for acid hydrolysis was as high as 40–64 % H_2_SO_4_ [[16], [17], [18],^38^]. However, the acid concentration used for hydrolysis in this study, 25 % H_2_SO_4_, was found to be a high concentration feasible for use with all cellulose parts obtained from OPTS because parenchyma cellulose obtained from OPTS-Pt was destroyed when acid concentration higher than 25 % H_2_SO_4_ was used for hydrolysis. The percentage of crystallinity of MCC obtained from OPTS in this study, which was approximately 60 %, was slightly lower than that reported by Lamaming et al. [18], which was approximately 64 % obtained from OPT. This might be because the concentration of sulfuric acid they used (64 %) was higher than that used in this study (25 %), therefore more amorphous content should be eliminated.

### 3.5 Powder properties of MCC derived from OPTS

Due to the undesirable appearance of MCC obtained from the vascular bundle parts of OPTS and the very low yield percentage of MCC obtained from the parenchyma top part, only MCC obtained from the parenchyma bottom and middle parts of OPTS were selected for this study, and next. The powder properties are presented in comparison to Comprecel® M102 in Table 2. The bulk densities of the two MCCs obtained from OPTS were relatively low compared to Comprecel® M102, with MCC-OPTS-Pm having a lower bulk density than MCC-OPTS-Pb. This was related to the highly porous texture, especially for MCC-OPTS-Pm. The low bulk density of OPTS-derived MCC might be because of the low density of OPT compared to hardwood. Moreover, this was because both MCCs were obtained from parenchyma sections, which are physically softer and more porous than from vascular bundles [17]. In addition, according to the density decrease with height of OPT, MCC obtained from two different sections (bottom *vs* middle OPTS) have different bulk densities. As cell walls thicken over time, they become more pronounced at the bottom than at the top. In upper oil palm trunks, the cells are younger and do not have thicker secondary cell walls, so the bulk density is correspondingly lower [44]. The tapped density also showed the same trend as the bulk density. A lower bulk density MCC would still yield a higher tapped volume due to the high porous nature of the substance, resulting in a lower tapped density.

**Table 2:** Powder properties of MCC obtained from bottom and middle sections of OPTS compared with Comprecel® 102.

Test	MCC-OPTS-Pb	MCC-OPTS-Pm	Comprecel® M102
Bulk density (g/cm^3^), n = 3	0.28 ± 0.00^b^	0.21 ± 0.00^c^	0.33 ± 0.00^a^
Tapped density (g/cm^3^), n = 3	0.33 ± 0.01^b^	0.26 ± 0.00^c^	0.41 ± 0.01^a^
True density (g/cm^3^), n = 3	1.41 ± 0.01^b^	1.39 ± 0.01^b^	1.49 ± 0.01^a^
Angle of repose (°), n = 3	32.16 ± 0.52^c^	35.81 ± 0.80^b^	43.97 ± 0.69^a^
Compressibility index	15.11	18.24	19.16
Hausner ratio	1.18	1.22	1.24
Degree of polymerization, n = 3	183.94 ± 0.27^c^	161.30 ± 0.10^b^	223.86 ± 0.18^a^
Residue on ignition (%), n = 3	0.06 ± 0.02^a^	0.06 ± 0.01^a^	0.02 ± 0.01^b^
pH, n = 3	5.94 ± 0.00^a^	5.93 ± 0.01^a^	5.74 ± 0.01^b^
Loss on drying (%), n = 3	6.04 ± 0.35^a^	6.06 ± 0.58^a^	5.11 ± 0.24^a^
Water soluble substances (%), n = 3	0.15 ± 0.03^a^	0.12 ± 0.02^ab^	0.09 ± 0.02^b^
Ether soluble substances (%), n = 3	0.01 ± 0.00^a^	0.01 ± 0.00^a^	0.01 ± 0.00^a^

Note: The same superscript letter in the same row indicates no statistical difference analyzed by One-Way ANOVA at the 0.05 significance level.

For the true density, there was no significant difference between the MCC obtained from OPTS-Pb and OPTS-Pm, however, their values differed from the true density of Comprecel® M102. The true density of cellulose should be between 1.582 and 1.599 g/cm^3^ [45]. However, the true density of MCC obtained from both OPTS-Pb and OPTS-Pm and Comprecel® M102 was lower. It is known that for cellulose with less than 100 % crystallinity, the true density may be lower than 1.582 g/cm^3^ [45]. The MCC obtained from OPTS-Pb and OPTS-Pm had a lower percentage of crystallinity than Comprecel® M102. This might be the reason for the lower true density. Moisture content is another important factor that can affect the true density of a substance because it can change the mass and volume of a substance [46]. Moisture can disrupt the cellulose chain interaction and can affect the swelling properties of cellulose [46]. As a result, the volume of the substance can be increased. It had been reported that moisture content higher than 5 % would result in a decrease in the true density. Commercial MCC has been reported a true density in the range of 1.42–1.46 g/cm^3^ at a typical moisture content of 4 % [45,46]. The relatively lower true density of OPTS-derived MCC found in this study might be a result of their lower crystallinity (approximately 60 %) and higher moisture content (approximately 7 %).

The flowability of MCC obtained from OPTS-Fb and OPTS-Fm was evaluated as the angle of repose, compressibility index, and Hausner ratio in comparison with Comprecel® M102, and the results are shown in Table 2. It was found that the MCC obtained from OPTS-Pb had better properties than the other two. The angle of repose value of MCC-OPTS-Pb as shown (32.16°) was classified as good flow according to USP criteria [21], while MCC-OPTS-Pm and Comprecel® M102 with a larger angle of repose values (35.81° and 43.97°) were classified as fair and passable flow, respectively. This property follows two other flow property parameters, compressibility index and Hausner ratio. From the values, it was indicated that MCC-OPTS-Pb had good flow, while MCC-OPTS-Pm and Comprecel ® M102 had fair flow. An important factor affecting the flow properties of powders is particle size. This might be the reason that the MCC obtained from OPTS, which has a higher particle size than Comprecel® M102, (as seen from SEM) has better flowability [47]. However, MCC-OPTS-Pm, although the particle size was higher than MCC-OPTS-Pb, the flowability was slightly worse. This might be because the surface of MCC-OPTS-Pm was slightly rougher and had lower bulk density than MCC-OPTS-Pb, thus hindering the powder flow [48,49]. The flowability of a powder is an important factor in determining how well it can flow during formulation preparation, especially for tablets or capsules. This could affect the uniformity of the dosage unit. MCC derived from OPTS, especially from OPTS-Pb, might have the potential to be used in the preparation of tablets by direct compression method. However, substances with lower flowability like MCC-OPTS-Pm could improve flowability, for example by preparing the powder into granules before tablet compression, called the wet granulation method [50].

The degree of polymerization (DP) of MCC obtained from OPTS-Pb and OPTS-Pm was found to be 183.94 and 161.30, respectively, which was lower than that of Comprecel® M102 (223.86). The DP of MCC derived from oil palm components has not been reported before. However, it was reported that the DP of MCC obtained from plant cellulose was in the range of 100–400 [51]. The important factors affecting the DP of MCC obtained from plant cellulose were the concentration and time used in the acid hydrolysis process [7]. High acid concentration or acid hydrolysis time usually increases crystallinity but usually reduces the yield and DP of MCC. The acid hydrolysis conditions employed in this study (25 % H_2_SO_4_, 1 h) were considered suitable because the crystallinity and yield of MCC were within the acceptable range, and the DP was also within the acceptance criteria, i.e., not more than 350, as specified in USP 2023 [23].

Other powder properties of MCC obtained from OPTS-Pb and OPTS-Pm performed in this study including residue on ignition, pH, loss on drying (LOD), water-soluble substances and ether-soluble substances were within the acceptance criteria as specified in USP 2023 [23]. The value of nearly zero percent residue on ignition results should indicate that the MCC prepared from both OPTS-Pb and OPTS-Pm were free from inorganic impurities. The pH values obtained should indicate that the final rinsing process with water was adequate, with a pH in the range of 5–7. LOD less than 7 % should be due to proper drying time and storage conditions. Finally, the results of nearly zero percent water and ether soluble substances should indicate that the prepared MCCs were free from residual organic impurities, both soluble and insoluble impurities.

### 3.6 Tablet compression properties of MCC derived from OPTS

**Fig. 5:**
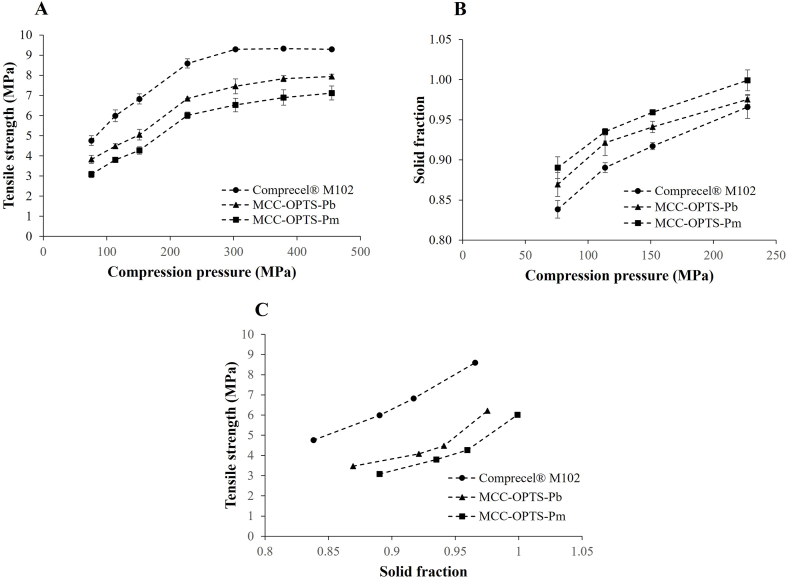
A: Tabletability profiles, B: Compressibility profiles, and C: Compactibility profiles of MCC obtained from bottom and middle sections of OPTS compared with Comprecel® M102.

The tablet compression properties of MCC obtained from OPTS-Pb and OPTS-Pm compared with Comprecel® M102 were presented as tabletability profile, compressibility profile, and compactibility profile as shown in Fig. 5. In the tabletability profile (Fig. 5A), which shows the relationship between the tablet compression pressure and the tablet tensile strength, the applied compression pressure ranged from 75 MPa to 455 MPa. The tensile strength increased relatively linearly with increasing compressive pressure initially in the range of 75 MPa–227 MPa. The tensile strength began to increase gradually after compression pressure greater than 227 MPa and did not increase further in the case of Comprecel® M102 or only slightly increased in the case of MCC obtained from OPTS-Pb and OPTS-Pm. This characteristic was obtained because MCC is a plastic deformation type pharmaceutical excipient and it exhibits plastic deformation characteristics, especially at high compression pressures [52,53], that is, when the compression pressure reaches a certain level (approximately 200 MPa), the tensile strength does not increase further even with increasing compression pressure. MCC obtained from OPTS-Pb and OPTS-Pm had lower tensile strength than Comprecel® M102 at the same compression pressure used. However, their application in tablet formulations should not be a problem as the tensile strength remained higher than the typical value required for tablets, i.e. 1 – 3 MPa, using typical compression pressure in the range of 100–150 MPa in pharmaceutical tablet preparation [54]. The lower tabletability of the MCC obtained from OPTS compared to Comprecel® M102 might be a result of particle size. A smaller particle size increased in tensile strength. The finer the MCC particles, which have a higher surface area, the closer they are to each other, so the adhesion between the particles is stronger when compressed, resulting in the tablet having a higher tensile strength [55]. Crystallinity might also affect the tensile strength of tablets. The tensile strength decreased with the decrease in MCC crystallinity [56]. Comprecel® M102, which had approximately 2 % higher crystallinity than MCC obtained from OPTS-Pb and OPTS-Pm, could then provide higher tensile strength. Another factor affecting the tabletability of MCC is bulk density, with lower bulk density MCC allowing for more tableting [7]. However, no such observation was found in this study that lower bulk density MCC-OPTS-Pm still presented less tabletability than higher density MCC-OPTS-Pb. Bulk density likely had less effect than particle size. The effect of bulk density would compensate for the effect of particle size, therefore MCC-OPTS-Pb still has slightly higher tabletability than MCC-OPTS-Pm.

For compressibility, the relationship between tablet compression pressure and tablet solid fraction is shown in Fig. 5B. It was found that the solid fraction increased as a function of compression pressure, which was similar in the three MCC types, but the degree of profile was different. The highest solid fraction was obtained in MCC-OPTS-Pm followed by that in MCC-Pb and the lowest in Comprecel® M102 along all compression pressures studied. Compressibility measures how easily a material changes volume when compressed. An important factor affecting compressibility is therefore density. MCC-OPT-Pm, which had the lowest density, the particles were most loosely assembled, resulting in the most compressible. The solid fraction of a tablet is the inverse of its porosity, i.e. tablets with a high solid fraction have a low porosity. The lower bulk density MCC-OPTS-Pm should have higher porosity and then facilitate higher compressibility than the higher bulk density MCC-OPTS-Pb. Too high a solid fraction can cause problems such as capping of the tablet and prolonging the dissolution. A maximum solid fraction for most pharmaceutical excipients is recommended at 0.95 [57]. MCC obtained from OPTS-Pb and Comprecel® M102 gave a solid fraction below 0.95 in the typical compression pressure range of 100–150 MPa, while MCC obtained from OPTS-Fm gave a solid fraction slightly above 0.95 at a compression pressure of 150 MPa. Therefore, if MCC obtained from OPTS-Pm is to be used, its compressive properties should be improved before use in tablet preparation, e.g., by mixing with other excipients.

For compactibility profiles of MCC obtained from OPTS-Pb and OPTS-Pm compared to Comprecel® M102, the results are shown in Fig. 5C. The tablets’ tensile strength of all three excipients increased exponentially as solid fraction increased. The compactibility behavior of the studied MCCs was consistent with the tabletability behavior. The highest compactibility was achieved by Comprecel® M102 followed by MCC-OPTS-Pb and MCC-OPTS-Pm, respectively. Size and density, which are inversely related, should be the main factors affecting compactibility as they affect tabletability [7,55]. MCC-OPTS-Pb, which had a higher density than MCC-OPTS-Pm, was less fluffy so its particles should be compacted together more efficiently when compressed to achieve the same solid fraction. Although the MCC obtained from OPTS had lower compactibility properties compared to the commercially available MCC, Comprecel® M102, however, at a lower solid fraction of 0.95, the tensile strength of the tablets obtained from both MCCs was still higher than the typical tensile strength of tablets (1–3 MPa) [54]. This result indicated that MCC obtained from OPTS-Pb and OPTS-Pm had the potential to be used as an excipient for tablets.

### 3.7 Elastic recovery of tablets prepared from OPTS-derived MCC

**Fig. 6:**
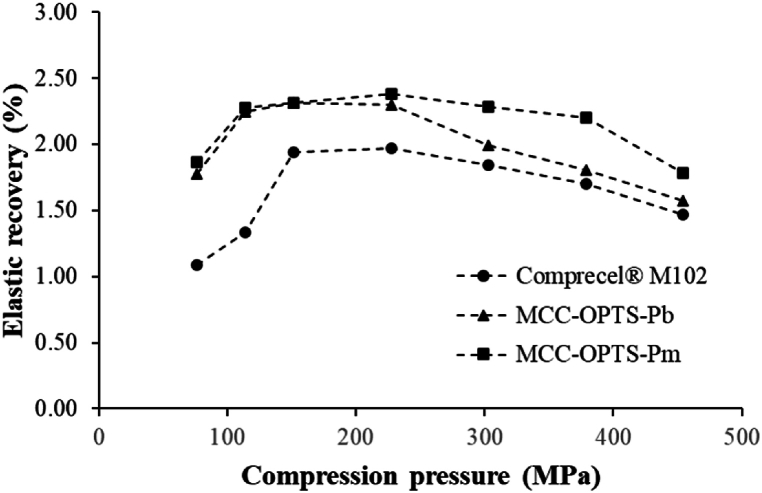
Elastic recovery of tablets prepared from MCC obtained from bottom and middle sections of OPTS compared with tablets prepared from Comprecel® M102.

The elastic recovery of tablets prepared from OPTS-derived MCC was studied to evaluate their elastic characteristics after compression at various compression pressures; the result is shown in Fig. 6. The elastic recovery could be observed in both MCC obtained from OPTS and comprecel® M102 but at relatively low as 1–2.5 %. This may be because MCC is a plastic deformation type excipient that is compacted at relatively high strength, especially at high compression pressure. Although highly plastic materials such as MCC exhibit plasticity, they also exhibit elasticity independently [52]. At compression pressures lower than 200 MPa, the elastic recovery increased with increasing compression pressure because the MCC particles were not fully compacted, causing tablet expansion. Comprecel® M102 can be compressed better than MCC obtained from OPTS due to its smaller particle size and higher density, resulting in lower elastic recovery. When compression pressures higher than 200 MPa were used, the elastic recovery of Comprecel® M102 and MCC-OPTS-Pb decreased with increasing compression pressure due to the better compactibility of these compounds compared to MCC-OPTS-Pm. MCC-OPTS-Pm, which had worse tabletability and compactibility, resulted in the highest elastic recovery among the three types of MCC comparisons. The results regarding tablet compression were negatively related to the expansion properties.

## 4 Conclusions

This study aimed to investigate the potential of cellulose extracted from oil palm trunk slabs (OPTS), residues from oil palm lumber processing for pharmaceutical tablets. The focus was on evaluating the suitability of microcrystalline cellulose extracted from different parts of OPTS with respect to trunk height (bottom, middle, and top parts) for tablet diluent application. Before the extraction process of cellulose, the vascular bundle and parenchyma tissue from each part were mechanically separated. The characteristics of the MCCs as well as the tablet's properties were then evaluated and compared with those of the commercial ones. The conclusions can be drawn as follows.

- The yield of MCC derived from both types of wood cells (vascular bundle and parenchyma tissue) decreased from the bottom (48.04 % from vascular bundle and 33.80 % from parenchyma tissue) to the top (40.49 % from vascular bundle and 8.35 % from parenchyma tissue) of the trunk height, in which the decreased MCC yield of parenchyma tissue was greater.
- MCC obtained from parenchyma tissue was more suitable for pharmaceutical tablets based on the texture (finer) and particle size (smaller), and therefore, MCC obtained from parenchyma tissue was used for further study, except the MCC from the top part was ignored due to its relatively low MCC yield.
- The density (bulk, tap, and true density), flowability, and degree of polymerization (DP) of MCC derived from the parenchyma tissue of the bottom part were greater than that of MCC derived from the parenchyma tissue of the middle part, while the crystallinity of MCC from both parts was not different. Compared with Comprecel® M102, however, the density, DP, and crystallinity of both MCCs were slightly lower. Other powder properties including residue on ignition, pH, loss on drying, water-soluble substances, and ether-soluble substances of both MCC obtained from OPTS were within the acceptance criteria of USP 2023.
- The tablet compression properties (tabletability, compressibility, and compactibility) of MCC obtained from parenchyma tissue of the bottom OPTS section were greater than those of the middle OPTS section due to greater MCC powder's properties but slightly lower than that of the Comprecel® M102 due to its lower bulk density and crystallinity and higher particle size. However, tablet compression properties of MCC obtained from the bottom OPTS section met the general requirement for tablet manufacturing, showing that it could be potentially used as tablet diluents.

## CRediT authorship contribution statement

**Chutima Jantarat:** Writing – review & editing, Writing – original draft, Visualization, Project administration, Investigation, Funding acquisition, Formal analysis, Data curation, Conceptualization. **Sirikanya Kaewpradit:** Writing – review & editing, Writing – original draft, Visualization, Methodology, Formal analysis. **Jiraporn Chingunpitak:** Writing – review & editing, Visualization, Methodology, Formal analysis. **Suthon Srivaro:** Writing – review & editing, Visualization, Methodology, Formal analysis.

## Data availability statement

Data will be made available on request.

## Funding statement

This work was supported by 10.13039/501100017170Thailand Science Research and Innovation Fund Contract No. FRB660041/0227.

## Declaration of competing interest

The authors declare the following financial interests/personal relationships which may be considered as potential competing interests: Chutima Jantarat reports financial support was provided by 10.13039/501100017170Thailand Science Research and Innovation. If there are other authors, they declare that they have no known competing financial interests or personal relationships that could have appeared to influence the work reported in this paper.
